# Wetlands of International Importance: Status, Threats, and Future Protection

**DOI:** 10.3390/ijerph16101818

**Published:** 2019-05-22

**Authors:** Ting Xu, Baisha Weng, Denghua Yan, Kun Wang, Xiangnan Li, Wuxia Bi, Meng Li, Xiangjun Cheng, Yinxue Liu

**Affiliations:** 1 State Key Laboratory of Simulation and Regulation of Water Cycle in River Basin, China Institute of Water Resources and Hydropower Research, Beijing 100038, China; xuting900515@163.com (T.X.); yandh@iwhr.com (D.Y.); pingguo88wangkun@163.com (K.W.); lixn0555@163.com (X.L.); 170201010001@hhu.edu.cn (W.B.); limenglee@126.com (M.L.); 2 College of Hydrology and Water Resources, Hohai University, Nanjing 210098, China; 3 Department of Hydraulic Engineering, Institute of Water Resources and Hydrology, Tsinghua University, Beijing 100084, China; 4 China Highway Engineering Consultants Corporation Data Co., Ltd., Beijing 100089, China; cheng_xiangjun@126.com; 5 School of Geographical Sciences, University of Bristol, Bristol BS8 1TH, UK; Yinxue.liu@bristol.ac.uk

**Keywords:** Wetlands of International Importance, Ramsar Sites, wetland degradation, wetland threat, wetland management, wetland protection

## Abstract

The 2303 Wetlands of International Importance distribute unevenly in different continents. Europe owns the largest number of sites, while Africa has the largest area of sites. More than half of the sites are affected by three or four impact factors (55%). The most significant impact factors are pollution (54%), biological resources use (53%), natural system modification (53%), and agriculture and aquaculture (42%). The main affected objects are land area and environment of the wetlands, occurred in 75% and 69% of the sites, respectively. The types most affected by land area occupation are river wetlands and lake wetlands, the types with the greatest impact on environment are marine/coastal wetlands and river wetlands, the type with the greatest impact on biodiversity is river wetlands, the types most affected by water resources regulation are marsh wetlands and river wetlands, and the types most affected by climate change are lake wetlands and marine/coastal wetlands. About one-third of the wetland sites have been artificially reconstructed. However, it is found that the proportions of natural wetland sites not affected or affected by only one factor are generally higher than that of wetland sites both containing natural wetlands and human-made wetlands, while the proportions of wetland sites both containing natural wetlands and human-made wetlands affected by three or four factors are generally higher than that of natural wetland sites. Wetland sites in the UK and Ireland are least affected among all countries. Wetland management plans in different regions still have large space for improvement, especially in Africa and Asia. The protection and restoration of global wetlands can be carried out in five aspects, including management and policy, monitoring, restoration, knowledge, and funding.

## 1. Introduction

Wetlands play an irreplaceable role in regulating the global climate, maintaining the global hydrological cycle, protecting the ecosystem diversity, and safeguarding human welfare [1,2]. Wetland ecosystems can not only bring indirect services to human beings, but also bring direct economic values to human beings [3,4]. The value per ha of wetland ecosystem services ranks first among all kinds of ecosystems, and the total values of wetland ecosystem services account for 47% of the values of the global ecosystem [3]. Therefore, it is one of the most important and productive ecosystems [4,5]. However, in past centuries, instead of attaching importance on wetlands, humans regarded wetlands as a harbor of mosquitoes, carriers of disease, and sources of death [6,7]. Therefore, settlers and early governments began to reclaim wetlands in large areas and make full potential use of the wetlands [7]. Due to the dual effects of human activities and natural factors, the wetland area in the world has been decreasing, and the wetland quality has been deteriorating [8,9,10,11,12,13]. Davidson gave an astonishing fact that wetlands around the world had degraded by about 87% since 1700 in data existing regions, and the degradation mainly occurred in the 20th and early 21st centuries [11]. Dugan [14] and OECD (Organization for Economic Co-operation and Development) [15] estimated that the world had lost 50% of its wetlands since 1900. Coincidentally, the Ramsar [16] and Mitsch et al. [5] also insisted that the world had lost more than half of the wetlands, and the loss mainly occurred in the 20th century. Ramsar Convention Secretariat reported a 35% reduction of global wetlands with data available between 1970 and 2015 [13]. Undoubtedly, this will also lead to a reduction in the value of wetland ecosystem services [12]. According to recent research of Costanza et al. [17], the value of marsh wetland ecosystem services decreased by 9.9 trillion dollars per year from 1997 to 2011, which was equivalent to 1.4 times of China’s GDP (Gross Domestic Product) in 2011 (if the exchange rate is 6.5). It is irrefutable that the degradation of wetlands will bring huge economic losses.

Since most of the services provided by wetland ecosystems have not been traded in the economic market, the value of wetland ecosystems continues to be neglected or underestimated by stakeholders, government, and public [18]. Wetlands are still facing the threat of loss or degradation [7,12]. Until recently, it has been recognized that wetlands not only contain the value of biodiversity and as habitats for plant animal and fish species, but also can bring many environmental services or functions [4,5,17,19,20,21,22,23]. Thus, wetland policy has begun to shift from encouraging development to protecting and rational utilization [24]. In the context of cognition transformation, the Ramsar Convention was signed in 1971 [4,24]. It is not only a global intergovernmental treaty of wetland conservation and wise use, but also the first environmental convention in the world [25,26]. Ramsar Convention aims at protecting wetland ecosystem function, maintaining wetland culture, and finally realizing sustainable socio-economic development through local, regional and national action and international cooperation [13,25,27]. Promoted by the Ramsar Convention, countries all over the world have participated in the research of wetland protection and wise use [13]. Now, the Ramsar List is the world’s largest network of protected areas [28]. By now, there are 2303 Wetlands of International Importance (Ramsar Sites) on the territories of 169 countries across the world, covering about 229 million ha [28], which accounts for about 19% of the global wetland area (which is recorded as 1210 million ha) [13,29].

Although there several positive news stories about the Ramsar Sites, they are still under threat [12,13]. In 2014, the NRSCC (National Remote Sensing Center of China) monitored 100 major Ramsar Sites around the world. They found that the total area of these wetlands decreased by less than 1% between 2001 to 2013, but the wetland ecosystems had degenerated [30]. According to the analysis results of Wetland Extend Trends (WET) index developed by UN WCMC (UN Environment World Conservation Monitoring Centre) [31], the indexes in the Ramsar regions all showed continuing progressive downward trends between 1970 to 2017 [13,32]. Moser et al. [33] showed that 84% of Ramsar’s listed wetlands had experienced or been threatened by ecological changes. Analysis of the major threats to wetlands in Asia [34] and the Neotropics [35] revealed that 85% and 81% of the wetland sites in Asia and Neotropics were mainly threatened by hunting, pollution, agricultural drainage, settlements, and urbanization [33]. A study by Kleijn et al. [36] suggested that it was unclear whether the increase of species abundance in Ramsar Sites was caused by conservation management or already existed before conservation designation. Guareschi et al. [37] analyzed 36 Ramsar Sites in southern Spain and found water-birds were not a reliable indicator of the status of wetland ecosystem health, which was consistent with the result of Kleijn et al. [36]. They also suggested that it is necessary to create a diversified and complete network to protect the wetland biodiversity. Finlayson [10] found ineffective management at a national level by analyzing the Contracting Parties` national reports. It can be concluded that the Ramsar Convention plays a positive role in wetland protection, but the protection and restoration of wetlands need to be further improved.

However, there are few studies relating to the status of the Ramsar Sites on a country or a region, and the results are mixed [12]. Little attention has been paid to the effectiveness of wetland conservation [38]. It is unknown whether these wetlands have been effectively protected after being added into the Ramsar List. In order to obtain the basic and synthesis information of the wetlands on the Ramsar List, a systematical survey on the distribution of the Ramsar Sites has been conducted. Meanwhile, the threat factors have also been analyzed to find out what are the main factors affecting the Wetlands of International Importance, distributions of different factors, and the main impact factors of different wetland types, which can indirectly reflect the status and future trends of global wetlands. In view of the continuous expansion of human-made wetlands, the impact differences between natural wetlands and wetlands containing artificial reconstruction have also been explored, which can provide some reference for wetland protection and restoration. Knowledge of the basic and synthesis information of the wetlands is very important for formulating appropriate policies and implementing priorities [39]. Finally, some suggestions for future wetland management and protection have been put forward according to the relevant information.

## 2. Data and Methods

### 2.1. Data

According to the paper drafting time, the deadline for data acquisition is 17th April 2018. The specific information of the 2303 wetlands was obtained from the Ramsar official website [28]. In order to express data sources simply, we just provide the basic web link as the data source, because the data collection and statistics process involves multiple web interface of each country in the Ramsar official website. Specific details need to be accessed to each country`s webpage on the basis of this webpage (for details, see Reference [28]). The authors classify the data and adjust some sites according to the geographic location. Other data sources are related to government reports and papers.

### 2.2. Classification of Wetland Impact Factors

Ramsar Convention classifies wetland impact factors into 12 categories, which can be divided into human factors and natural factors, as listed in Table 1 [28]. According to the affected objects, the human activities factors are mainly divided into land area impact (including the land occupation of agriculture and aquaculture, natural system modifications, human settlements, and transportation and service corridors), environment impact (including the influence from pollution, human intrusions and disturbance, and energy production and mining), biodiversity impact (including overuse of biological resources use and invasive and other problematic species and genes), and water resources impact (mainly including water regulation) (Table 1). The natural factors include climate change and severe weather impact and geological events impact [28].

In order to further qualitatively analyze the extent to which wetlands are affected by human factors, the authors simply classify the types of wetlands affected by human factors. In this study, the authors use the number of impact factors to indirectly reflect the extent of wetland impact. Generally, the more factors affecting wetlands, the greater the threat to wetlands. Although this method cannot quantitatively reflect the extent of wetland impacts, to a certain extent, it can reflect the probability of wetland threat. Because of the data restrictions, the authors classify the wetlands simply. According to the above four human factors, wetlands that have no main human factors are divided into level 0, and wetlands that are affected by one factor are divided into level 1. By analogy, they are divided into 0 to 4 levels. The classification results are shown in Table 2.

## 3. Distributions of the Ramsar Sites

Ramsar Convention divides wetlands into inland wetlands, marine/coastal wetlands, and human-made wetlands [13,28,40]. The inland wetlands include natural inland wetland sites and the sites both containing natural inland wetlands and human-made wetlands. While the marine/coastal wetlands include natural marine/coastal wetland sites and the sites both containing natural marine/coastal wetlands and human-made wetlands. Ramsar Convention also classifies the above three wetland types into 43 subtypes [28,39]. However, it is very difficult to obtain the relevant information of these subtypes separately [39]. In order to facilitate the follow-up research of inland wetlands, this study further subdivides inland wetlands into river wetlands, lake wetlands, and marsh wetlands based on the references of the Ramsar Convention, China Forestry Bureau, and wetland researchers [25,28,41].

**Figure 1:**
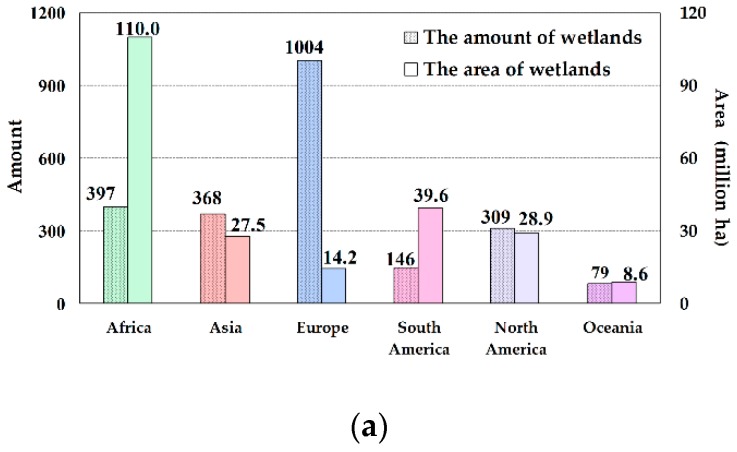

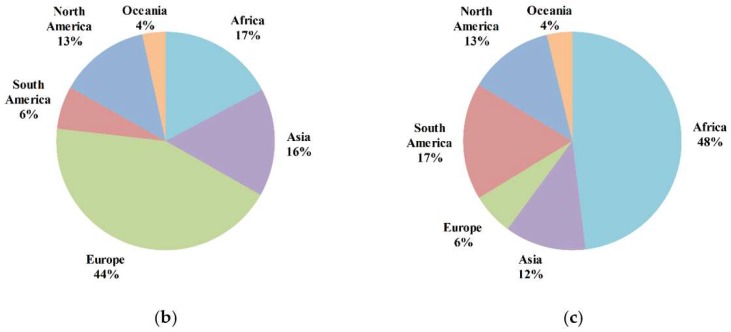
The amount and area (**a**), amount ratio (**b**), and area ratio (**c**) of the Ramsar Sites in each continent. The data source of Figure 1 is the Ramsar official website. For details, see Reference [28].

The 2303 Ramsar Sites distribute unevenly in different continents. Europe has the largest number of sites, the total amount is 1004, occupying 44% of all Ramsar Sites (Figure 1). But the area of these sites occupies just 6% of the total area. Although the amounts of the sites in Africa (397) and South America (146) account for only 17% and 6% of the total sites, respectively. However, due to the larger area of individual wetlands, the area of these sites account for 48% (110.0 million ha) and 17% (39.6 million ha) of the total area, respectively (Figure 1). The amount ratios of the sites in Asia (368) and North America (309) are 16% and 13%, respectively. And their area ratios are 12% (27.5 million ha) and 13% (28.9 million ha), respectively. Oceania has the least wetland sites, with a total of 79, occupying only 4% of the all, and the area ratio is also 4% (8.6 million ha). According to the wetland area of each continent in the latest published papers and reports, protected area of the six continents listed on the Ramsar Convention accounted for 92% (Africa), 7% (Asia), 9% (Europe), 21% (South America), 9% (North America), and 25% (Oceania) of the total wetland area on each continent, respectively [13,29].

Countries with the most sites are the United Kingdom (174 sites, accounting for 7.6% of the total sites) and Mexico (142 sites, accounting for 6.2% of the total sites), but the total area of them are 1.3 million ha (accounting for just 0.6% of the total area) and 8.7 million ha (accounting for 3.8% of the total area), respectively. Bolivia has the largest area under the Ramsar Convention`s protection, which is mainly distributed between 10°S and 20°S, with a total area of 14.8 million ha (accounting for 6.5% of the total area, while the amount proportion is just 0.5%); Countries with a protected area exceeding 10.0 million ha also include Congo, Canada, Chad, the Democratic Republic of Congo, and the Russian Federation, which are mainly distributed between 10°S–20°N and 40°N–60°N. The single sites with the largest protection area are the Ngiri-Tumba-Maindombe (a lake wetland from the Democratic Republic of Congo, with an area of 6.6 million ha, accounting for 2.9% of the total area) and the Queen Maud Gulf (a coastal wetland from Canada, with an area of 6.3 million ha, accounting for 2.7% of the total area). Sites with a protected area exceeding 5.0 million ha also include the Grands affluents (a river wetland from Congo, with an area of 5.9 million ha), the Sudd (a marsh wetland from South Sudan, with an area of 5.7 million ha), and the Okavango Delta System (a river wetland from Botswana, with an area of 5.5 million ha). Due to different protection purposes, four wetlands with an area of only one hectare have been added into the Ramsar List, which are the Ganghwa Maehwamareum Habitat in the Republic of Korea, the Somerset Long Bay Pond in the UK, the Ile Alcatraz in Guinea, and the Mare Aux Cochons high altitude freshwater wetlands in Seychelles.

**Figure 2:**
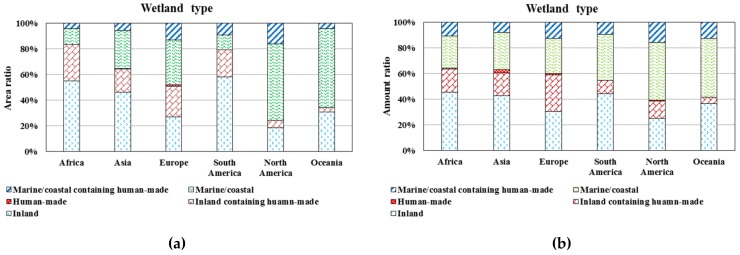
The area ratio (**a**) and amount ratio (**b**) of different wetland types in each continent. The data source of Figure 2 is the Ramsar official website. For details, see Reference [28].

**Figure 3:**
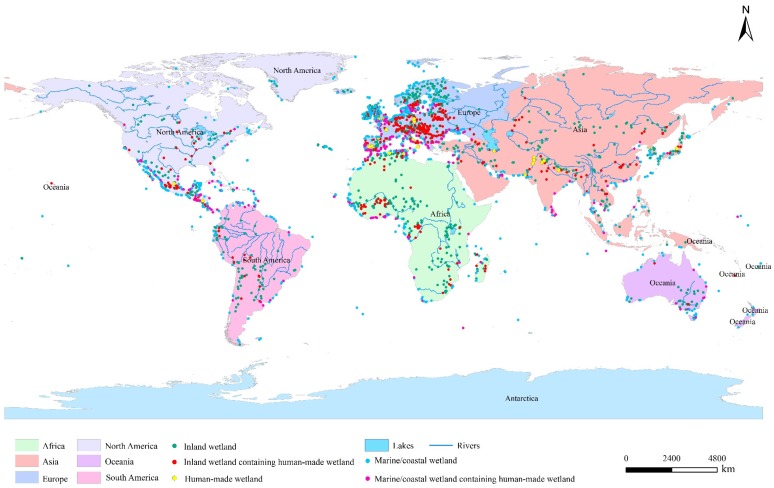
The distribution map of the Ramsar Sites. By referring to the free data obtained from the Ramsar’s official website, the authors use the Arcgis 10.2 software (ESRI (Environmental Systems Research Institute, Inc.), Redlands, CA, USA) to draw Figure 3. The data source is Reference [28].

The distribution of different types of wetlands is discrepant in the six continents. For inland wetlands, river wetlands account for the largest area proportion of 44%, and lake wetlands and marsh wetlands account for 27% and 29%, respectively. Inland wetlands mainly distribute in Africa (58% of the total area, of which 56% is river wetlands, 23% is lake wetlands, and 21% is marsh wetlands) (Figure 2a), which are primarily located in the Okavango Delta and the flood plain of Niger River, Zambezi River, Orange River, Nile River, etc. (Figure 3). Human-made wetlands are mainly reservoirs, most of which distribute in Asia (Pakistan and India) and Europe (UK and France). Marine/coastal wetlands mainly distribute in North America (Mexico) and Africa (the countries along the Atlantic coast) (Figure 3). There are 37.0% of the inland wetland sites and 28% of the marine/coastal wetland sites have been artificially reconstructed. The inland wetland sites containing human-made wetland sites mainly distribute in Europe (59.0%, primarily in the countries located in West and South Europe), Africa (15.0%, primarily in Congo, Burkina Faso, and Algeria), and Asia (13.3%, primarily along the Yangtze River). The marine/coastal wetland sites containing human-made wetland sites are mainly located in Europe (46.9%), North America (18.0%), Africa (15.4%), and Asia (11.0%) (Figure 2b). Noting that 414 wetland sites are distributed in high altitude areas, of which, the main types are alpine marsh wetlands (43%) and alpine lake wetlands (37%), mainly distributed in Africa (31%, the Atlas Mountains and the southern mountain region), Europe (29%, the Alps and the Iberian Peninsula), and Asia (21%, the Himalayas and the Mongolia Plateau).

## 4. Threats to the Ramsar Sites

Impact factors of wetland can be divided into wetland transformation and wetland destruction [5]. The degradation of most wetlands is affected by destructed factors, such as agriculture, infrastructure development, water use, pollution, etc. [13,40,42,43]. By 1985, it was estimated that 56% to 65% of wetlands in Europe and North America were drained for agriculture, which in Asia, South America, and Africa were 27%, 6%, and 2%, respectively [4]. Total loss of wetlands used for agriculture in the world accounted for 26% [15,44,45]. In the first 50 years of the 20th century, the loss of wetlands occurred mainly in Europe and North America. Since the 1950s, wetlands in tropical and subtropical regions have been increasingly degraded or lost, due to the conversion to agriculture use [15,46]. The use of water resources is another major cause of inland wetland degradation. To meet the growing demand for irrigation and hydropower, many rivers around the world are strictly controlled by dams [44]. There are 37% of the world’s 227 main rivers have been seriously affected, and 23% have been moderately affected [47]. There are still many rivers around the world threatened by large dams under construction or planned construction, especially in developing regions, such as the Yangtze River Basin in China, the La Plata Basin in South America, and the Tigris and Euphrates River Basin in the Middle East [47]. Taub [48] reported that, because of the increasing water demand, all rivers in Japan had built artificial lakes. Ironically, due to the silting problems, these lakes had lost 80% of their capacity. Pollution caused by population growth and socio-economic development is also a major factor in leading to wetland degradation and loss [13,49,50,51,52,53,54]. Eventually, the water resources shortage and pollution will bring about threats like wetland ecosystem degradation and species invasion [55].

A Ramsar Site means a wetland reserve. The area of a Ramsar Site and the actual area of wetland in a site are two different concepts. In general, the area of a Ramsar Site will not change unless it is expanded, adjusted, or merged with other protected areas [38]. The wetland loss mentioned here refers to the actual area loss of wetland, which can be affected by human activities and climate change [13,47]. Despite the concerted efforts of local, regional and national actions and international cooperation to protect these wetlands, human activities and climate change continue to have a significant influence on these wetlands as a result of economic development, population growth, and climate warming, etc. [12,13]. Because of the limitations of data acquisition at present, the authors use the incidence of impact factors to reflect the threats of various factors to these wetlands. Although the incidence cannot fully quantify the impact extent of each factor on wetlands, it can reflect the overall status of each factor in all wetlands to a certain extent. Here, the authors do not analyze in depth how each factor affects the wetlands and how quantitative the impact is, which will be the research directions in the future.

### 4.1. Threats to All the Ramsar Sites

#### 4.1.1. Distributions of the Threats

**Figure 4:**
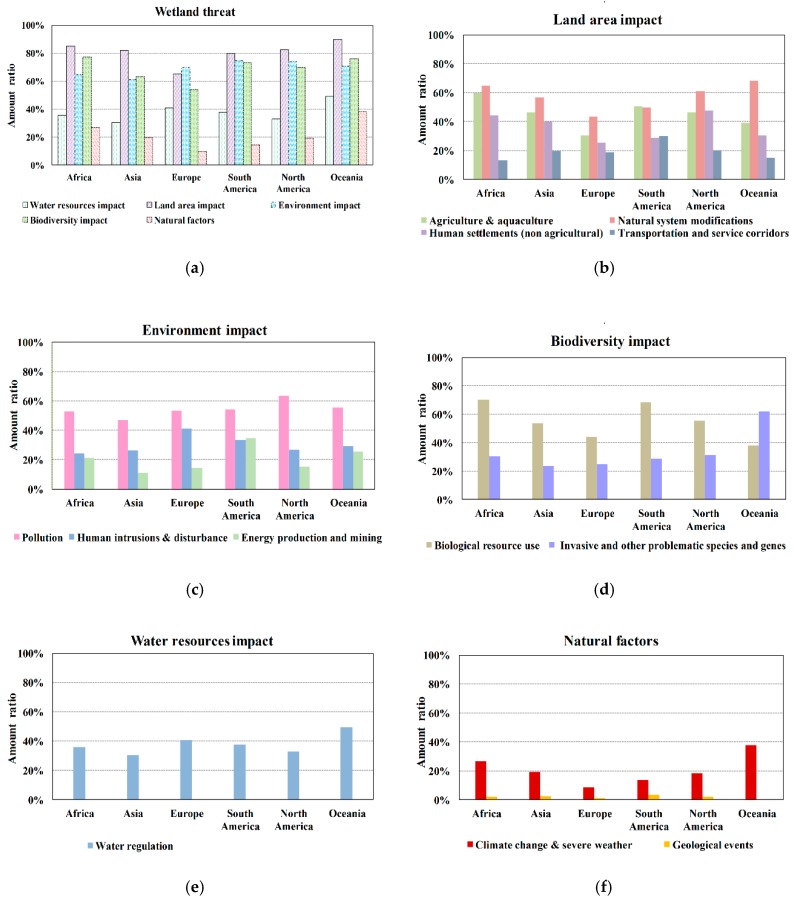
The amount ratios of impact factors to Ramsar Sites in each continent; (**a**) is for total impact factors; (**b**) is for land area impact; (**c**) is for environment impact; and (**d**) is for biodiversity impact; (**e**) is for water resources impact; and (**f**) is for natural factors.

Among different factors, the impact on the land area is the most serious, occurred in 75% of the sites. The percentages of affected wetland sites in five continents (except Europe) are all over 80%, and that of Europe is as high as 65% (Figure 4a). The impact on the land area is mainly reflected in the occupation of wetland area by agriculture and aquaculture, natural system modifications (including vegetation clearance/land conversion and dams and water management/use), human settlements, and transportation and service corridors construction. Among them, the impact of natural system modifications is the most serious, occurred in 53% of the sites, followed by the impact of agriculture and aquaculture (42%), and human settlements (34%) (Figure 4b). Regions with greater impact of natural system modification land encroachment are Oceania (68%, mainly distributed in the regions along the southeast coasts and the Darling River), Africa (65%, mainly distributed in the regions along the west and southeast coasts, the Victoria Lake, the Tanganyika Lake, the Chad Lake, the Niger River, and the Zambezi River), and North America (61%, mainly distributed in the southwest coasts of Mexico). Regions with greater impact of agriculture and aquaculture land occupation are Africa (60%, mainly distributed in the regions along the north coasts and the Niger River), South Africa (51%, mainly distributed in the regions along the west coasts), North America (46%, mainly distributed in the southwest coasts of Mexico), and Asia (46%, mainly distributed in the regions along the Indus River, the Ganges River, and the northeast coasts). The regions more seriously affected by human settlement are North America (48%, mainly distributed in the southwest coasts of Mexico), Africa (44%, mainly distributed in the regions along the northwest coasts), and Asia (40%, mainly distributed in the regions along the Indus River, the south and east coasts). And the regions more seriously affected by transportation and service corridors are South America (30%, mainly distributed in the regions along the west coasts).

The impact on the environment is also a major factor, especially in South America and North America, where 75% and 74% of the wetland sites are affected, respectively. At the same time, the amount ratios of the sites whose environment have been affected in the other four continents are all more than 60% (Figure 4a). The impact on the environment is mainly caused by pollutants coming from household sewage, urban waste water, garbage and solid waste, agricultural and forestry effluents, and industrial effluents, etc. The amount ratio of wetland sites affected by pollutants in all continents is about 54% (ranking the top of all impact factors), of which, North America is the largest (63%, mainly distributed in the southwest coasts of Mexico) (Figure 4c). The human intrusions and disturbance of recreational and tourism activities will also bring impact to the wetland environment. The most serious regions are Europe and South America, with ratios of 41% (mainly distributed in all the coastal regions and the regions along the Danube River and Rhine River) and 34% (mainly distributed in the regions along the northwest coasts), respectively. There are also some regions where energy production and mining activities have an impact on the wetland environment, mainly distributed in South America (with the amount ratio of 35%, mainly located in the regions along the west coasts).

A myriad of wetland sites is also affected by biodiversity resources overuse, especially in Africa, South America, and North America, with amount ratios of 71%, 68%, and 56%, respectively (Figure 4a). Invasive and other problematic species and genes is also a major impact, with 62% of the wetlands in Oceania have affected by it (Figure 4d).

Data analysis shows that the water resources of these wetlands in all continents have been impacted. The percentage of impacted wetlands ranges from 30% to 50% (Figure 4a,e), especially in Oceania (mainly distributed in the regions along the Murray River) and Europe (mainly distributed in the regions along the Rhine River, the Danube River, the Vanern Lake, and most coasts). Water resources are intercepted in 50% of the wetlands in Oceania, which may be related to the distribution pattern of water resources in Australia. Australia is the continent with the least precipitation. Although the water resources per capita in Australia is large, the total water resources of it are less. Due to the extremely uneven spatial and temporal distribution of precipitation in Australia, coupled with the increasing water consumption brought by population growth, a large number of water conservancy projects need to be built to regulate water storage [56]. As a result, the natural water source of the wetland was intercepted, and the ecological water source of the wetland was reduced. It can be noted that 40.6% of the wetland sites in Europe also suffered from water resources being impacted. The increasing water use of agriculture and tourism in Europe leads to the shortage of water resources, and the continuous over-withdrawal of water resources of rivers, lakes, and wetlands, resulting in the lack of ecological water [57].

Some wetlands are affected by climate change and extreme weather, geological disasters, etc., of which, climate change and extreme weather are the main nature factors [58,59]. The most affected type is marine/coastal wetlands (41%), followed by lake wetlands (24%) and marsh wetlands (23%) (Figure 4f). From latitude zoning, the most influential region is 40°S–50°S (56% of the wetland sites in this area have been affected by climate change and severe weather, it is mainly distributed in the southeast area of Oceania.), followed by 10°S–20°S (28%, it is mainly distributed in the north regions of Oceania and the southeast areas of Africa.), 10°N–20°N (28%, it is mainly distributed in the northeast regions of Africa and Mexico), 20°S–30°S (20%), and 30°N–40°N (19%, it is mainly distributed in Central Asia, the Tibetan Plateau regions of China and the coastal regions of Japan, and the arid desert climate zones in northern Africa). The most affected continent is Oceania, of which, 30 out of 72 wetlands are affected by climate change and extreme weather. The largest amount of wetland sites affected by climate change and severe weather is Africa (105), followed by Europe (85) and Asia (71). Wetlands affected by geological events are mainly marine/coastal wetlands (62%) and inland alpine wetlands (26%), which may be caused by seabed and mountain geological activities.

From the perspective of wetland types, the types most affected by land area occupation are river wetlands (87%) and lake wetlands (80%), the types with greatest impact on the environment are marine/coastal wetlands (72%) and river wetlands (70%), the type with the greatest impact on biodiversity is river wetlands (79%), the types most affected by water resources regulation are marsh wetlands (45%) and river wetlands (42%), and the types most affected by climate change are lake wetlands (19%) and marine/coastal wetlands (15%).

Remarkably, 63% of the sites in the United Kingdom and 44% of the sites in Ireland have not been affected, and 24% of the sites in the United Kingdom and 49% of the sites in Ireland are threatened by only one or two factors. The water regulation and pollution are the main factors affecting wetlands in the United Kingdom, while pollution and agriculture land use are the main factors affecting wetlands in Ireland. Wetlands sites in the United Kingdom and Ireland are least affected in all countries.

#### 4.1.2. Levels of the Impacted Wetlands

**Figure 5:**
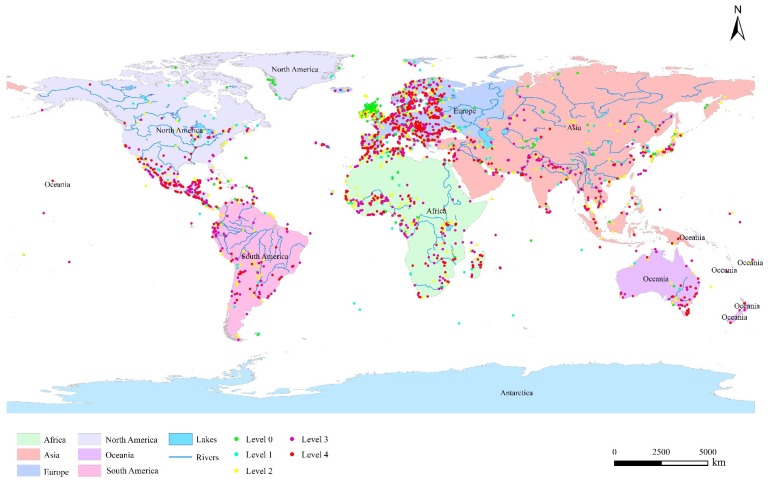
The distribution of the sites with different levels. By referring to the free data obtained from the Ramsar’s official website and conducting relevant calculations and statistics, the authors use the Arcgis 10.2 software to draw Figure 5. The data source is Reference [28].

From the classification results, more than half of the sites affected by three or four impact factors (55%), of which Oceania is the most serious, with level 3 and 4 accounting for 68%, followed by North America (63%) and South America (63%). The proportions of level 3 and 4 of other continents are 60% in Africa, 51% in Asia, and 50% in Europe (Table 2). The unaffected sites (level 0) mainly distributed in the United Kingdom and Ireland (Figure 5). The sites affected by one impact factors (level 1) mainly located in the United Kingdom, Ireland, Spain, and Denmark in Europe, Togo in Africa, China and South Korea in Asia, Canada and Mexico in North America (Figure 5), and the sites affected by three or four impact factors scattered in each continent (Figure 5).

About one-third of the wetland sites have been artificially reconstructed, which may have adverse effects on wetland ecological functions. The classification results show that the proportions of natural wetland sites not affected or affected by only one factor are generally higher than that of the sites both containing natural wetlands and human-made wetlands (11% for inland wetlands and 12% for marine/coastal wetlands). The proportion differences in each continent are 9% for inland wetlands and 11% for marine/coastal wetlands in Africa, 18% for inland wetlands and 12% for marine/coastal wetlands in Asia, 17% for inland wetlands and 16% for marine/coastal wetlands in Europe, 14% for marine/coastal wetlands in South America, 6% for inland wetlands and 16% for marine/coastal wetlands in North America, and 10% for inland wetlands in Oceania (Table 2). The proportions of the sites both containing natural wetlands and human-made wetlands affected by three or four factors are generally higher than that of natural wetland sites (16% for inland wetlands, and 19% for marine/coastal wetlands). The proportion differences in each continent are 8% for inland wetlands and 25% for marine/coastal wetlands in Africa, 26% for inland wetlands and 12% for marine/coastal wetlands in Asia, 20% for inland wetlands and 22% for marine/coastal wetlands in Europe, 6% for inland wetlands and 20% for marine/coastal wetlands in South America, 11% for inland wetlands and 22% for marine/coastal wetlands in North America, and 18% for marine/coastal wetlands in Oceania (Table 2).

However, it is noteworthy that wetland conservation in China is in a transitional stage, and wetland restoration and reconstruction projects have been vigorously carried out in recent years. Forty-two percent of the inland wetland sites and 38% of the marine/coastal wetland sites have been artificially reconstructed or altered (which are all higher than the global average). Among the 20 typical Ramsar Sites in China, monitored by the NRSCC, the area of human-made wetlands accounts for as much as 50%, which has significant limitations on maintaining the overall ecological function of wetlands [30].

### 4.2. Degradation and Threats of the Typical Ramsar Sites

Due to the limitations of data acquisition, here, the authors mainly discuss the degradation and threats of the wetlands in typical countries and typical wetland areas.

#### 4.2.1. Asia

In 2014, the NRSCC has monitored 20 Ramsar Sites in Asia. They found that the total area of these 20 wetland sites decreased by about 1% between 2001 and 2013, the total water area and the landscape integrity also showed decreasing trends, and the wetland ecosystems had degenerated, which was closely related to insufficient water supply and climate change [30]. The Sanjiang Plain is China’s largest natural marsh wetland distribution areas [60]. From 1954 to 2015, due to agricultural reclamation, the area of wetlands in Sanjiang Plain had dropped dramatically by 79.4% (about 2.99 million ha) [61]. In the eight wetland sites in Sanjiang Plain monitored by the NRSCC, the area of these wetlands also decreased by 33,600 ha between 2001 and 2013. The main reason for the wetland loss was the transformation of natural wetlands to agricultural land and constructed wetlands, while the main reason for wetland degradation was the reduction of water supply [30]. Lake Urmia, one of the largest permanent high salinity lakes in the world, whose area decreased by 40% between 2001 and 2013, while 49% of flooded wetlands were converted to artificial surfaces or bare land [30]. The Mekong River basin includes many Ramsar Sites in South Asian countries. The lack of coordination in river basin management in these countries, especially in the wetland management of the Mekong Delta region, is a major concern. In recent years, the wetlands in the Mekong River Basin have been threatened by many factors, such as agricultural intensification, urbanization, and industrialization. The most significant factor is the construction of dams and reservoirs [5].

#### 4.2.2. North America

Everglades National Park is the largest Ramsar Site of primitive wetland in the United States. About half of the original Everglades has disappeared, mainly due to the agriculture in the north and urban development in the east and west [5]. In recent years, a series of protection measures aiming at restoring natural water flow has been implemented in this area, and satisfying results have been achieved [30]. San Francisco Bay is recognized as one of the most important ecological estuaries in North America and one of the most altered and urbanized wetland areas in the United States. Since the first European settlers arrived, almost 95% of the wetlands have been destroyed. In order to develop agriculture and salt industry, they invaded the wetland first, then removed the vegetation, built dikes to drain the wetland, and eventually caused rapid degradation of the wetlands. Now, the deposition and erosion caused by upstream hydraulic mining are also one of the biggest threats to the wetlands [5]. However, the wetland ecosystems in Queen Maud Bay, distributed in the polar tundra with less human disturbance, have also deteriorated, suggesting that climate change is the main impact factor [30].

#### 4.2.3. South America

In NRSCC`s research, the total wetland area of 20 Ramsar Sites in South America has decreased by 0.26 million ha between 2001 to 2013, mainly in marshes and lakes. The degeneration of the wetland ecosystems and the transformation of swamps into artificial surfaces or bare land in these wetland areas, indicating that the main impact factors are human agricultural activities and precipitation changes [30]. The Lake Mar Chiquita is one of the largest saline lakes in the world. Its area decreased by 26%, and dry land area increased more than twice between 2001 and 2013. Agricultural reclamation was the main factor and water shortage was the biggest problem [30]. Pantanal is one of the largest wetland distribution regions in the world, which contains many inland wetland types of Ramsar Sites of South America. The Pantanal Matogrossense is a part of the largest, a permanent freshwater wetland in the Western Hemisphere [62]. With the development of the upper Paraguay River, Pantanal has been threatened by many elements, including the encroachment of pastoral and agricultural land use, water pollution caused by mining activities and agriculture, and the invasion of alien toxic species. Because of Pantanal’s remoteness, it has become a place for illegal wildlife trafficking and cocaine smuggling, where wetland management is difficult and expensive [5].

#### 4.2.4. Europe

In NRSCC`s research, the total wetland area of 10 Ramsar Sites in Europe has decreased by 3% between 2001 to 2013, mainly in marshes and lakes. Twelve percent of the reservoirs and 34% of the seasonal marshes have degraded into non-wetlands, and the interference degradation index has increased significantly, which may be related to long-term development in Europe [30]. The Lake Sevan is one of the world’s largest alpine freshwater lakes. Between 2001 to 2013, the agricultural areas in the region increased by 10%. And excessive groundwater extraction posed a huge threat to the lakes. Although the lakes have been artificially replenished since 2001, 17% of the forests and shrubs have been submerged, due to the replenishment. On the contrary, it has brought about ecological problems such as organic pollution and fish reduction [30]. The Danube Delta is one of Europe’s largest and most natural inland deltas, containing some of Europe’s inland wetland types of Ramsar Sites, which are degraded by drainage and activities related to agricultural development, gravel mining, and dumping [5,30,63]. Although major international studies on restoration have now been carried out in the delta, most of the recovery is simple, just restoring natural hydrology by dams and reconnecting waterways [5]. The Volga Delta, one of the largest inland deltas in the world, is on the edge of the Caspian Sea. The wetland is now being affected by a number of impact factors, including dam damage to the river’s natural hydrology, pollution from heavy industry and agriculture, and the decline of the Caspian Sea level [5,63]. The Wadden Sea, which contains many coastal wetland types of Ramsar Sites of Western Europe, is considered to be the most important coastal wetlands in Western Europe. However, a myriad of wetlands has been cultivated by local residents over the past few centuries [5].

#### 4.2.5. Africa

In NRSCC`s research, the total wetland area of 30 Ramsar Sites in Africa has decreased just two thousand hectares, however, wetland ecosystem degeneration is more serious. Seventeen percent of the river wetlands and 20% of the inland flood wetlands have degraded into non-wetlands, and many other wetland types have also degraded. The disturbance degradation index in Africa was high and kept constantly increasing [30]. The Lake Chad, an African transnational lake, suffered a 9% drop in lake surface and an 89% drop in seasonal herbaceous swamps between 2001 to 2013, which mainly due to the drought climate and drainage irrigation [30]. The signal site with the largest area is the Ngiri-Tumba-Maindombe, located in the Democratic Republic of Congo (DRC), Africa [28]. Now, the Ngiri-Tumba-Maindombe is under apparent threat because of the pressures from the rapidly growing population and illegal activities [62]. The DRC is one of the countries with the largest area of Ramsar Sites distributed in the world [28]. The Ramsar Sites in DRC play a vital role in the conservation of rare and endangered flora and fauna of the region. These are some of the last remaining sites in the country where human intervention and exploitation of natural resources are not allowed. However, years of civil war and political unrest in the country have adversely affected these natural habitats and flora, where animals are suffering from illegal human activities. Therefore, there is a need for urgent international attention to protect these fragile habitats [62]. The Okavango Delta System is one of the largest Ramsar wetlands in the world [28]. However, Okavango faces many threats now, mainly from increased burning (Okavango’s fire is natural), as well as upstream countries` intercepting water resources, tourism threats, raw materials overuse, etc. [5]. The Mangrove Swamps in West Africa Mangrove is distributed many coastal wetland sites of Ramsar. Now, the mangrove wetlands in these areas are being eroded by desertification, due to the reduction of persistent rivers caused by drought [5]. At the same time, it is also threatened by over exploitation and conversion to rice fields [5].

#### 4.2.6. Oceania

The NRSCC found that the wetland area changes in the two Ramsar Sites in Oceania, they monitored, were large. Between 2001 and 2013, the two wetlands reduced by 80,000 hectares, and 90% of the seasonal marshes were degraded into forest shrubs. The disturbance degradation index of Oceania was the highest among all continents [30]. The Kakadu National Park is the largest national park in Australia. Between 2001 to 2013, a large number of wetland ecosystems had degenerated, and some wetlands had been converted into artificial surfaces or bare land [30]. The Shoalwater and Corio Bays are the largest areas in east Queensland. The site is essential because of the only remaining remnant of its type, size, and condition in central Queensland. The main threats to the region include pollution, erosion, pests, and inappropriate recreational use [64]. The Whangamarino is the second largest peat bog and swamp complex on the North Island. The main threats to the peat bog are the reduction of river flooding, the deposition of silt caused by agricultural development, the increase of fire frequency, and the invasion of alien species [5].

From the above cases, we can conclude that these typical wetland sites all have suffered area loss and ecosystem degeneration. Human activities are the main impact factors, and climate change also has a certain impact on wetlands.

## 5. Management Plan of the Ramsar Sites

Wetland management in different regions of the world has different meanings in different periods. In the 1950s, most management decision-makers considered wetland management as wetland drainage [5]. Subsequently, with the efforts of a few supporters who deemed wetlands as habitats for wildlife, wetland management has evolved into a whole science about maintaining special hydrological conditions and optimizing fish and waterfowl populations [5,65]. It was not until 1975, that the concept of wetland management was added to the concept of flood control, coastal protection, and water quality improvement. Today, the meaning of wetland management is to set management objectives, which depends on the priorities of wetland managers, current environmental regulations, and the wishes of the many stakeholders [5]. The Ramsar Convention holds that wetland management implies the need to understand the past and present human use of wetlands, their current or future impacts, and ways to achieve optimal (sustainable) wetland use [66]. In the past 40 years, under the guidance of wetland management, wetlands in many developed areas have been protected or even restored. However, in some developing areas, there are few laws and restrictions on wetland protection and restoration [5]. In order to maintain the biodiversity and productivity of wetland ecosystems and allow their resources to be used wisely, centralized management actions are urgently needed to protect them [66]. A wetland management plan is indispensable for wetland conservation, including identifying the objectives of site management and the existing or potential impact factors, resolving conflicts, identifying and describing the actions needed to achieve management objectives, defining the monitoring requirements, maintaining continuity of effective management, helping obtain funding, achieving communication within sites, organizations, and stakeholders, demonstrating the effectiveness of management, and ensuring compliance with local, national, and international policies [66].

**Figure 6:**
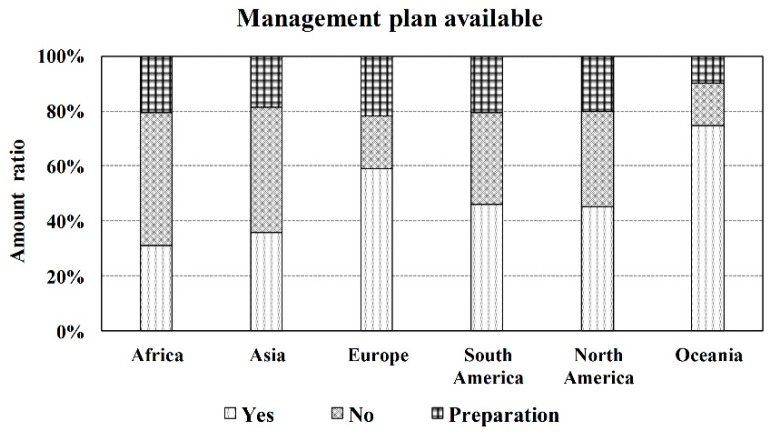
Amount ratio of management plan available of the Ramsar Sites in each continent. The data source of Figure 6 is the Ramsar official website. For details, see Reference [28].

The establishment of the Ramsar Convention has brought benefits to many wetlands. However, wetland management plans in most regions are not perfect, especially in Africa and Asia (Figure 6). In Africa, up to 49% of the wetland sites do not have management plans, while 20% of the sites are in preparation for management plans, and only 31% of the wetland sites have management plans. Similarly, the situation in Asia is not optimistic. Forty-five percent of the wetland sites in Asia have no management plans, 19% of Asian wetland sites are preparing management plans, and only 36% of Asian wetland sites have management plans now. More than half of wetland sites in South America and North America have yet to complete wetland management plans. The management plans in Oceania and Europe are relatively well developed. Only 15% and 19% of wetland sites have not yet formulated wetland management plans, while 10% and 22% of sites are preparing management plans, respectively.

Despite the fact that most wetland sites in Oceania and Europe have established management plans, they are still threatened by many factors. Up to 41% of the wetland sites that have developed the management plans are threatened by more than four impact factors. Therefore, it is uncertain whether these management plans have been effective in wetland conservation and restoration [65]. The inefficiency of wetland management plans may be due to the lack of participation of government, non-governmental, or other community organizations in the formulation processes [4,65]. The implementation of the wetland management plans may be more effective with the assistance of management agencies or actors, including government, non-governmental, and community organizations [4].

As wetlands in the UK are the least affected, the authors have studied its wetland management and found that the characteristics of wetland management in the UK can be summarized as: Firstly, it combined the comprehensive management system of watershed water resources; secondly, it implemented the wetland nature reserve system and established the nature reserve network; thirdly, it implemented the public purchasing system to purchase the private ownership wetland for better management and protection; and fourthly, it clarified the responsibilities and obligations of wetland owners to protect wetlands in the form of management agreements system [67].

## 6. Recommendation for Conservation and Restoration of Global Wetlands

Over the past 40 years, wetland conservation and restoration in developed countries have been progressing, but many developing regions have no regulations or restrictions to control the continuous destruction and pollution of wetlands [5]. Wetland ecosystems in these regions failed to play an essential role in maintaining ecological security, food security, freshwater security, and climate security [5]. It is imperative for local, regional and national actions and international cooperation to work together continually to strengthen global wetland conservation and restoration. Base on the successful experiences of various countries and suggestions from relevant scientists, the protection and restoration of global wetlands can be carried out in the following five aspects [13,25,41,68]:

### 6.1. Management and Policy

Wetland management can respond to the five kinds of threats to wetlands separately. For the impact on wetland area, in order to control the total area of wetland strictly, wetland area limits can be set. As protected areas are the most effective way to protect wetland [38], in the regions, where natural wetlands are widely distributed and rare and endangered species are concentrated, nature reserves can be set up. In the areas, where wetland resources need to be both protected and rationally utilized, wetland parks can be built. In the areas, where with protection or less area of wetland elements, wetland protection zones can be established. In addition, wetlands can be protected by setting up forest parks, scenic spots, water conservancy scenic spots, water source protection zones, and coastal parks, etc. For the impact on wetland environment, the limits of the pollution can be set to strictly control the total amount of pollutants discharged into the wetland. This can be achieved by combining wetland management with river basin environmental management. The discharge of pollutants from the agriculture, industry, animal husbandry, and domestic sewage, etc. around the wetlands, rivers, lakes, and seas should be strictly controlled. For the impact on wetland biodiversity, the limits of species diversity can be set. It is important to ensure the number of rare animals does not decrease and to assist them to reproduce. For the impact on wetland water resources, the limits of wetland ecological water can be set. This can be achieved by combining wetland management with river basin water resources management. By strictly controlling water intake from rivers and lakes, the ecological water quantity of wetlands can be guaranteed. For the impact of climate change on wetlands, climate prediction studies should be done ahead of time. In view of the long-term drought climate, the ecological water quantity of wetlands should be guaranteed in advance, and the ecological water supply should be implemented in time in the water-deficient areas. In order to avoid the secondary ecological impact of long-term precipitation on wetland overflooding, wetland drainage should be implemented in time.

To ensure the smooth implementation of wetland management, more scientific laws, regulations, and policies on wetland protection should be issued quickly to crack down and punish the wetland destruction. As the national conditions of each country and region are quite different, each country and region should issue laws, regulations, and policies in accordance with the local development.

### 6.2. Monitoring

It should be done to establish a global wetland dynamic monitoring system to assess the global wetland status, which is essential to wetland management and conservation [30,41]. It can be conducted by means of remote sensing and field observation. Remote sensing has been proved to be an effective technology for monitoring global wetlands [46,69,70], which can efficiently, accurately, and objectively monitor the spatial and temporal distributions and dynamic changes of wetlands on a large scale [30]. Field observation is an important means to reveal the changes in the structure and function of wetland ecosystems [41]. For remote sensing technology, it can continuously improve the remote sensing technology of wetland monitoring, and increase the resolution of monitoring data and the speed of data updating. The experience of the NRSCC can be referenced. It is the first case in the world to monitor and analyze the status and changes of large-scale Wetlands of International Importance using remote sensing technology [30]. For field observation, it can call on countries or regions around the world to regularly carry out domestic or regional wetland resource surveys and timely grasp the dynamic changes of wetland ecosystems. The implementation of field observation mainly includes the establishment of monitoring points, monitoring stations, and monitoring centers, the construction of the monitoring database and wetland information management platform [41]. At the same time, the monitoring results should be evaluated to provide timely reference information for wetland protection and restoration.

### 6.3. Restoration

Wetland restoration mainly includes the restoration or reconstruction of degraded or disappeared wetlands through ecological technology and ecological engineering [25]. In the process of wetland restoration, attention should be paid to respecting the natural attributes of wetland ecosystems, avoiding excessive human interference with the primitive wetlands, so that they can take advantage of their self-recovery ability [5,25]. In view of the different main impact factors of different wetland types, it is necessary to formulate corresponding restoration strategies according to the specific conditions of different wetland types. For river wetlands, they are greatly influenced by four kinds of factors of human activities. Measures that can be implemented include dredging the rivers, reducing pollution sources, increasing pollutant purification zones, controlling biodiversity, increasing ecological water quantity, etc. For lake wetlands, they are greatly affected by land occupation and climate change. Measures that can be implemented include returning farmland to the lakes, water diversion projects to ensure ecological water quantity, etc. For marsh wetlands, their biodiversity is most affected. The measures that can be implemented are to strictly prohibit the harvesting of biological resources, and priority should be given to the restoration of wetlands with important ecological protection objectives, such as migratory bird habitats, rare and endangered bird habitats. For marine/coastal wetlands, they are greatly impacted by pollution and climate change. Projects that can be implemented include controlling pollution sources, migrating eutrophic sediments, etc.

In recent years, China has implemented a large number of wetland restoration projects throughout the country, especially in the Source Regions of Three Rivers. After implementing a series of projects of ecological water replenishment, the ecological restoration effect is remarkable. The area of most lakes has increased, and the quality of most rivers has improved significantly. As the Qianhu wetland, at the source of the Yellow River, began to recover, the ecological characteristics and functional integration of the wetland have been enhanced obviously.

### 6.4. Knowledge

It is necessary to strengthen the public’s awareness of wetland resources protection and resource distress by education and enhance the awareness of wetland protection of global citizens, and expand wetland protection to the global scope [41]. It can be carried out in the following aspects: Launch wetland protection activities on World Wetland Day, Bird Week, and Wildlife Protection Month, etc.; publicize wetland protection awareness through the media; establish a wetland science education base in the wetland reserve; and add relevant knowledge of wetland protection to primary school textbooks. At the same time, it is indispensable to carry out wetland related scientific research. Scientific researches related to wetland protection and restoration should be encouraged and developed, as scientific research can improve wetland management and protection to a higher level. Scientific research on key technologies of wetland protection and restoration should be carried out to serve large-scale ecological restoration projects of wetlands. Finally, a new science, Wetland Science, can be developed in universities or scientific research institutions in the regions where wetland science research is relatively backward or lacking, attracting more scholars to participate in wetland conservation research.

### 6.5. Funding

Ensuring the source of funding for wetland conservation and restoration is the precondition for the above work to proceed successfully. The funding sources can be the support of the government or public welfare organizations, the ecological compensation from the surrounding areas for the impact of wetlands, and the public donations, etc. At the same time, without damaging wetlands, financial channels can be developed to guide communities towards organic agriculture and ecotourism. For example, eco-pastures in degraded wetlands can be planted with aquatic plants with higher economic value, such as water chestnut, water lily canopy, and so on. They can not only purify water quality, but also obtain economic sources, thus ensuring the financial chain of wetland conservation and restoration.

## 7. Conclusions

Although each continent has a certain number of wetlands signed with the Ramsar Convention, the total area of these wetlands accounts for less than 19% of global wetlands. Therefore, the Ramsar Convention calls for more wetlands around the world to join in the protection of it.

Among different factors, the impact on the land area is the most serious, mainly comes from the land occupation of natural system modification and agriculture. The most seriously impacted regions are mainly located along the coasts, major inland rivers, and lakes of Oceania and Africa. The impact on the environment is also a major factor, especially along the west coasts of South America and the southeastern coasts of Mexico. Sites with the greatest impact on water resources are located along major rivers, lakes, and coasts in Oceania and Europe. Sites affected by climate change and extreme weather are mainly marine/coastal wetlands, followed by lake wetlands and marsh wetlands, mainly located in the southeast area of Oceania and the northwest area of Africa. From different types, river wetlands are susceptible to the land occupation, environmental pollution, species invasion, and excessive regulation of water resources, which may be related to people’s preference for living near rivers since ancient times. Lake wetlands are vulnerable to land occupation and climate change, which may be due to the geographical environment of the lake and precipitation as the main source of the water supply of lakes. Biological resources of marsh wetlands are most affected, which may be due to the most abundant biological resources in marsh wetlands have attracted over-exploitation by human beings. Marine/coastal wetlands are most affected by pollution and climate change, which may be due to the economic development of coastal cities and sea level rise caused by climate warming.

According to the level classification results, wetland sites both containing natural wetlands and human-made wetlands are more susceptible to multiple factors. We can generally draw the conclusion that wetlands artificially reconstructed are more vulnerable, humans must follow the natural law in the process of wetland protection and restoration. More than one-third of the sites in China have been artificially reconstructed or altered, and the area of human-made wetlands at 20 monitoring points of the NRSCC`s research in China accounted for more than 50%. Whether it will bring adverse effects is worth further investigation. 

Wetland management plans in each continent are not perfect, especially in Africa and Asia. Despite most wetland sites in Oceania and Europe have established management plans, they are still threatened by many factors. It is imperative for local, regional and national actions and international cooperation to work together continually to strengthen global wetland conservation and restoration, so that the implementation of the wetland management plans may be more effective. It can be carried out in five aspects, including management and policy, monitoring, restoration, knowledge, and funding.

Because of the data restrictions, this study only makes a simple assessment and analysis of the status of the Ramsar Sites, and the level classification of affected wetland sites is also relatively simple. In future research, it is necessary to continue to accumulate effective data, so as to classify the levels more scientifically and conduct more accurate and in-depth research on how these factors affect wetlands.

## Figures and Tables

**Table 1:** Classification of wetland impact factors.

Class	Subclass	Subdivision
Human factors	Land area impact	Agriculture and aquaculture
		Natural system modifications
		Human settlements (non-agricultural)
		Transportation and service corridors
	Environment impact	Pollution
		Human intrusions and disturbance
		Energy production and mining
	Biodiversity impact	Biological resource use
		Invasive and other problematic species and genes
	Water resources impact	Water regulation
Natural factors	-	Climate change and severe weather
	-	Geological events

The data source of Table 1 is the Ramsar official website. For details, see Reference [28].

**Table 2:** The levels of the wetland sites in each continent (%).

Continent	Wetland Type	Subdivision	Level of the Wetland Sites (%)					Continent	Wetland Type	Subdivision	Level of the Wetland Sites (%)				
			0	1	2	3	4				0	1	2	3	4
Africa	Inland wetland	Natural	6	10	27	38	19	South America	Inland wetland	Natural	2	5	26	45	23
		Non-natural	1	6	28	35	31			Non-natural	7	7	13	47	27
	Marine/coastal wetland	Natural	4	19	23	39	15		Marine/coastal wetland	Natural	10	12	27	40	12
		Non-natural	2	10	10	45	33			Non-natural	0	7	21	36	36
	Sub-total		4	12	24	39	21		Sub-total		4	8	25	42	21
Asia	Inland wetland	Natural	11	20	26	29	15	North America	Inland wetland	Natural	5	13	17	44	21
		Non-natural	3	9	20	47	22			Non-natural	0	12	12	33	43
	Marine/coastal wetland	Natural	10	8	33	31	17		Marine/coastal wetland	Natural	12	12	22	39	15
		Non-natural	0	7	33	40	20			Non-natural	2	6	16	41	35
	Sub-total		8	13	27	35	17		Sub-total		7	12	18	40	23
Europe	Inland wetland	Natural	17	13	24	29	17	Oceania	Inland wetland	Natural	3	7	31	31	28
		Non-natural	7	6	19	29	37			Non-natural	0	0	50	50	0
	Marine/coastal wetland	Natural	25	15	24	27	10		Marine/coastal wetland	Natural	0	6	22	39	33
		Non-natural	9	14	20	29	30			Non-natural	10	0	0	70	20
	Sub-total		16	12	22	28	22		Sub-total		3	5	24	41	28
Total	Inland wetland	Natural	11	13	25	34	18	-	-	-	-	-	-	-	-
		Non-natural	5	7	20	33	34			-	-	-	-	-	-
	Marine/coastal wetland	Natural	15	13	25	33	14		-	-	-	-	-	-	-
		Non-natural	5	10	18	37	30			-	-	-	-	-	-
	Total		10	11	23	34	21		-	-	-	-	-	-	-

Natural means natural inland wetlands or natural marine/coastal wetlands. Non-natural means inland wetland sites both containing natural inland wetlands and human-made wetlands; or means marine/coastal wetland sites both containing natural marine/coastal wetlands and human-made wetlands. The unit of level 0 to 4 is %, which represent the wetland amount ratios of different levels.
